# Effect of Electroacupuncture Versus Cognitive Behavioral Therapy for Perimenopausal Insomnia: Protocol for a Noninferiority Randomized Controlled Trial

**DOI:** 10.2196/51767

**Published:** 2023-11-09

**Authors:** Huixian Wang, Xintong Yu, Jing Hu, Yanting Zheng, Jia Hu, Xuqiu Sun, Ying Ren, Yunfei Chen

**Affiliations:** 1 Department of Acupuncture and Moxibustion Yueyang Hospital of Integrated Traditional Chinese Medicine and Western Medicine Shanghai University of Traditional Chinese Medicine Shanghai China; 2 Acupuncture Anesthesia Clinical Research Institute Yueyang Hospital of Integrated Traditional Chinese Medicine and Western Medicine Shanghai University of Traditional Chinese Medicine Shanghai China; 3 Shanghai Yangpu Mental Health Center Shanghai China

**Keywords:** perimenopausal insomnia, acupuncture, electroacupuncture, cognitive behavioral therapy, randomized controlled trial, CBT, sleep disorder, insomnia, perimenoupause, effectiveness

## Abstract

**Background:**

Perimenopausal insomnia (PMI) has a high global incidence, which is common in middle-aged women and is more severe than nonmenopausal insomnia. Effective treatments with fewer side effects and more consistent repeatable results are needed. Acupuncture, a therapy based on traditional Chinese medicine, is safe and may be effective for PMI. It is widely accepted in Western countries, and evidence supports the use of acupuncture as a main or supplementary therapy. Cognitive behavioral therapy is also used to improve sleep quality. It has structured sessions and has been recommended as a first-line treatment for insomnia (cognitive behavioral therapy for insomnia [CBT-I]) by the American Association of Physicians. However, few randomized controlled trials have been conducted to compare the effectiveness of these 2 therapies. This study will be performed in perimenopausal women with insomnia to determine the efficacy of electroacupuncture (EA) versus CBT-I.

**Objective:**

This study aimed to compare the preliminary effectiveness and safety of EA and CBT-I for PMI through a randomized controlled noninferiority study design.

**Methods:**

This study is designed as an assessor-blinded, noninferiority, randomized controlled trial. A total of 160 eligible participants with PMI will be randomly divided into 2 groups to receive either EA or CBT-I. Participants in the EA group will receive electroacupuncture for 8 weeks. The intervention will be delivered 3 times weekly for a total of 12 sessions and 2 times weekly for the next 4 weeks. Meanwhile, participants in the control group will undergo CBT-I (once a week) for 8 weeks. Treatment will use 7 main acupoints (GV20, DU24, EX-HN3, EX-HN18, EX-CA1, RN6, and RN4) and an extra 4 acupoints based on syndrome differentiation. The primary outcome is the Insomnia Severity Index. The secondary outcome measures are the Pittsburgh Sleep Quality Index; Menopause-Specific Quality of Life; Menopause Rating Scale; Hamilton Depression Scale; Hamilton Anxiety Scale; hot flash score; and the level of estradiol, follicle-stimulating hormone, and luteinizing hormone in serum. Sleep architecture will be assessed using polysomnograms.

**Results:**

Participants are currently being recruited. The first participant was enrolled in January 2023, marking the initiation of the recruitment phase. The recruitment process is expected to continue until January 2025, at which point data collection will commence.

**Conclusions:**

This trial represents a pioneering effort to investigate the efficacy and safety of EA and CBT-I as interventions for PMI. It is noteworthy that this study is conducted solely within a single center and involves Chinese participants, which is a limitation. Nonetheless, the findings of this study are expected to contribute valuable insights for clinicians engaged in the management of PMI.

**Trial Registration:**

Chinese Clinical Trial Registry ChiCTR2300070981; https://www.chictr.org.cn/showprojEN.html?proj=194561

**International Registered Report Identifier (IRRID):**

DERR1-10.2196/51767

## Introduction

Perimenopause is the period from the onset of menstrual irregularities to 1 year after amenorrhea has occurred and encompasses the physiological changes that occur before and after a woman stops menstruating [1]. During this period, women are prone to perimenopausal insomnia (PMI) due to fluctuations in estradiol, progesterone, and follicle-stimulating hormones that disrupt the central nervous control of the sleep-wake state [2]. The incidence of sleep disorders in perimenopausal women ranges from 43% to 48% [3], and in the United States, the incidence is 31% to 42% [4]. PMI seriously affects the quality of life and physical and mental health of perimenopausal women and is associated with the development of many diseases such as low immunity, diabetes, obesity, inflammation development, cardiovascular disease, mood disorders, cognitive dysfunction, and cancer [5-9].

In addition to PMI, perimenopausal syndromes are usually managed with menopausal hormone therapy (MHT) [10-12]. However, some patients are reluctant to choose MHT due to adverse effects [13], such as increasing the risk of uterine fibroids, breast cancer, and thrombosis [14,15]. The main reason for the increased incidence of breast cancer in patients taking hormones is related to the use of synthetic progestins such as dydrogesterone and 17α-hydroxyprogesterone derivatives in MHT regimens [16,17].

Cognitive behavioral therapy (CBT), as the first-line nonpharmacological treatment for insomnia, is an appropriate treatment for PMI without any concerns of drug-related side effects [18,19]. The focus is primarily on sleep problems, but CBT also targets stress, low mood, and vasomotor symptoms. Convincing effects of CBT for insomnia (CBT-I) in menopausal women have been found [20-22]. However, due to its limited availability in the community, the limitations of the psychological expertise of the operator, the high treatment costs, and the cognitive level of the patient, CBT has not been widely implemented in China [23]. It is, therefore, essential to develop treatment strategies to help patients manage PMI and improve their quality of life.

Acupuncture is widely practiced in the prevention and treatment of PMI [24] and is effective in the treatment of PMI and various sleep disorders through significantly reducing Pittsburgh Sleep Quality Index (PSQI) and Insomnia Severity Index (ISI) scores [25-27], improving sleep efficiency and overall nighttime sleep time in insomnia patients and reducing night-time wakefulness. Acupuncture is also a safe and effective treatment for perimenopausal depression with short- to medium-term efficacy [28].

Electroacupuncture (EA) therapy is a product of the combination of modern electrical stimulation and acupuncture therapy, with both stimulation effects, and can effectively and safely improve the sleep quality and quality of life of patients with PMI. Compared to CBT, EA is less expensive, simpler to administer, easier to promote, and has a high level of acceptability and enjoyment [29,30]. Despite the positive effects of EA on improving sleep, no studies have compared EA with CBT in women with PMI. We aimed to compare the effect of CBT-I versus EA for PMI and simultaneously evaluate the safety and acceptance of EA. We hypothesized that EA would not be inferior to CBT in patients with PMI.

## Methods

### Study Design

This study is an assessor-blinded, randomized controlled trial. Participants will be randomized into the EA group or the control group in a 1:1 ratio. After the baseline assessment and informed consent has been obtained, a randomization number will be sent to the doctor. The statisticians and outcome assessors will be blinded to the allocation.

**Figure 1:**
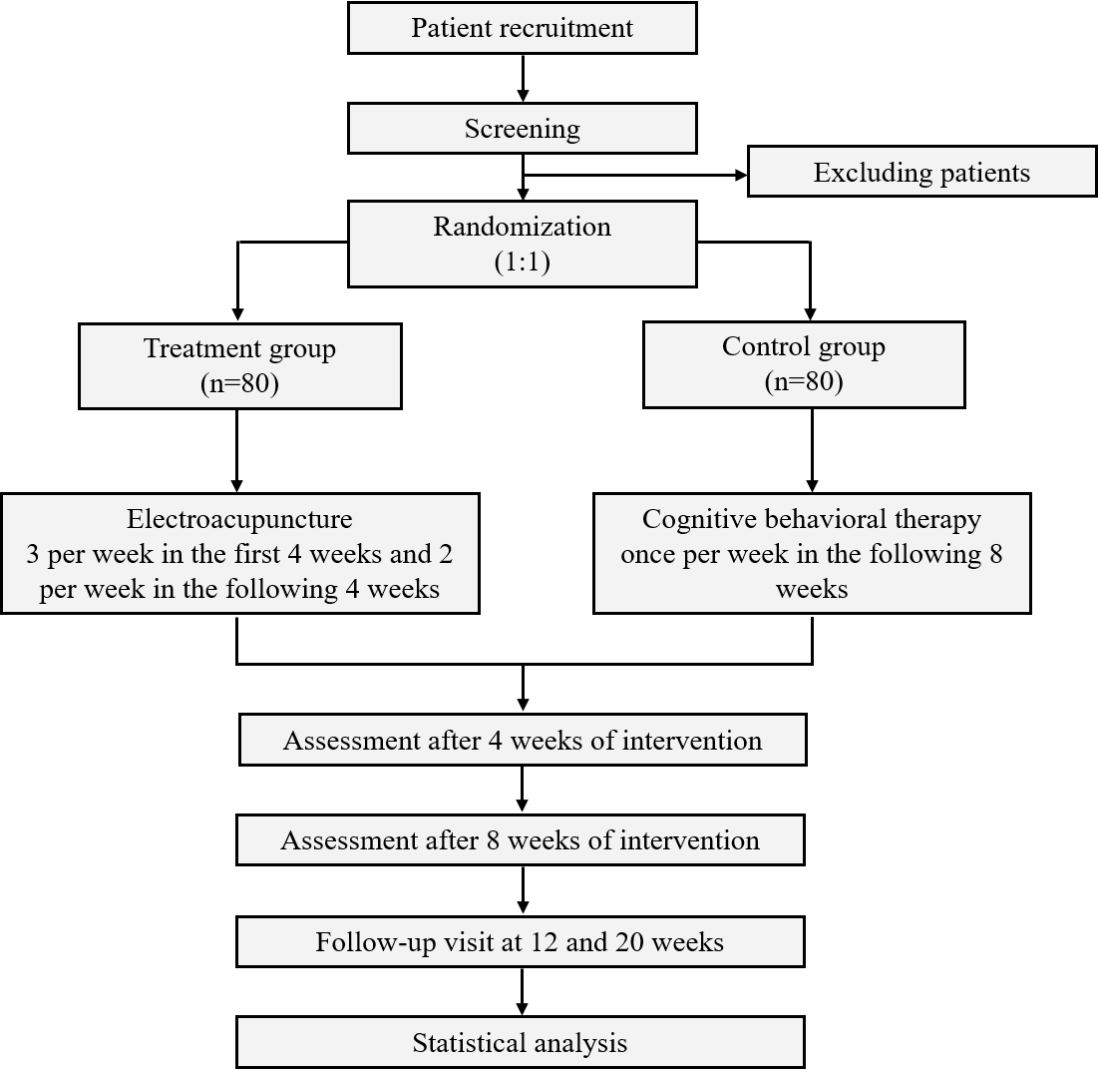
Flow diagram on the randomization of participants into the electroacupuncture and cognitive behavioral therapy groups.

The duration of the trial is 21 weeks (Figure 1), including a 1-week baseline assessment period (weeks 0-1), an 8-week long treatment period (weeks 1-8), and a 12-week-long follow-up period (weeks 9-21) (Table 1). The trial will be conducted in accordance with the principles of the Declaration of Helsinki and has been approved by the ethics committee of the participating hospitals. The standard protocol items adhere to the Recommendations for Interventional Trials [31] and the checklist of SPIRIT (Standards for Reporting Interventions in Clinical Trials of Acupuncture) is presented in Multimedia Appendix 1).

**Table 1:** Schedule of enrollment, interventions, and assessments for the electroacupuncture versus cognitive behavioral therapy study.

Schedule			Study period and time points (weeks)					
			Recruitment	Baseline	Treatment		Follow-up	
			1	0	4	8	12	20
**Enrollment**								
	Eligibility screen		✓					
	Informed consent form		✓					
	Medical history		✓					
	Allocation			✓				
**Interventions**								
	Electroacupuncture		—^a^	—^a^	—^a^	—^a^	—^a^	—^a^
	Cognitive behavioral therapy		—^b^	—^b^	—^b^	—^b^	—^b^	—^b^
**Assessments**								
	**Primary outcome**							
		ISI^c^		✓	✓	✓	✓	✓
	**Secondary outcomes**							
		MENQOL^d^		✓	✓	✓	✓	✓
		PSQI^e^		✓		✓		
		MRS^f^		✓		✓		
		HAMA^g^		✓		✓		
		HAMD^h^		✓		✓		
		Hot flashes		✓		✓		
		PSG^i^		✓		✓		
		Serum sex hormone		✓		✓		
	**Others**							
		Adverse events			✓	✓	✓	✓
		Patient compliance			✓	✓	✓	✓

^a^Patients in the treatment group will be treated with electroacupuncture for a total of 20 times.

^b^Patients in the control group will be treated with cognitive behavioral therapy for a total of 8 sessions.

^c^ISI: Insomnia Severity Index.

^d^MENQOL: Menopause-Specific Quality of Life.

^e^PSQI: Pittsburgh Sleep Quality Index.

^f^MRS: Menopause Rating Scale.

^g^HAMA: Hamilton Anxiety Scale.

^h^HAMD: Hamilton Depression Scale.

^i^PSG: polysomnograms.

### Recruitment

This trial will be conducted at Yueyang Hospital of Integrated Traditional Chinese and Western Medicine affiliated with Shanghai University of Traditional Chinese Medicine from January 2023 to January 2025. In total, 160 participants will be recruited through posters, newspapers, and hospital websites. We will publish some advertisements in WeChat through the subscriptions of the acupuncture and gynecology departments. To reach the target sample size, lectures to publicize the project program will be given regularly.

Patients who sign up will initially be screened by phone and then asked to participate in a face-to-face interview to confirm whether they meet the criteria and to conduct further surveys. Written informed consent will be obtained before baseline outcome assessments and data collection.

### Participants

The sample size is based on the ISI. After 8 weeks of intervention, the mean ISI score baseline changes are approximately –7.7 (4.18) [21,32]. Assuming that the ISI score of the acupuncture group is slightly lower than that of the CBT group after 8 weeks of treatment, the ISI score should be no more than –2, with a noninferior effect threshold of –4 [33]—assuming that the single-side significant level is α=.025, power of the trial is 1 – β=.80, and the ratio between the EA group and the CBT-I group is 1:1. As calculated by PASS software (IBM Corp), each group requires 71 cases. Assuming that the dropout rate is 10%, the final sample size should be 160.

Eligible patients will be randomly assigned to either the EA or CBT-I group and will receive EA treatment or CBT-I consultation, respectively. The inclusion criteria are the International Classification of Sleep Disorders (Third Edition) criteria for insomnia disorder; women aged 40-60 years; according to STRAW+10 (Stages of Reproductive Aging Workshop + 10), signs of menopausal tendency that began to appear from clinical features, endocrinology, and biology until 1 year after final menopause; insomnia lasting for >3 months; total ISI score >14; and willingness to participate in the investigation and provide written informed consent prior to the trial.

The exclusion criteria are the use of menopause hormone therapy and sedative or antianxiety drugs; nonnatural menopause (due to surgery, radiation, and medication); any diseases that cause sleep disturbance; unknown causes of vaginal hemorrhage; diagnosis of mental disorders or any serious physical illness; receipt of CBT or acupuncture for insomnia; participation in any other clinical trial within the last 6 months; and pregnancy or lactation.

### Randomization and Blinding

This study uses a randomized block design. Individual random numbers will be generated with SAS (version 9.4; SAS Institute). The random table is reproducible, and the random seeds will be recorded in the file. A researcher who will not interact with the participants during the recruitment or interventions will take charge of using sealed opaque envelopes to keep the randomized allocation sequence. The trial is designed to be open-ended, but the evaluators will be blinded to patient group assignments. The therapists who administer the treatment are not involved in the evaluation of the trial.

### Control Design

An EA (treatment) group and a CBT (control) group will be set up. Parallel controls will be performed between the 2 groups. The CBT group will undergo first-line treatment recommended by the guidelines, and this study has a noninferiority design. The purpose of this clinical study will be achieved by comparisons with first-line therapy.

### Interventions

The acupuncturist and psychologist have qualifications and 10 years of clinical work experience. All study participants will receive 7 days of training prior to the trial to familiarize them with the process. The therapist will send a message from a phone account assigned to the study to confirm the session 1 day in advance.

### Treatment

Treatment group participants will receive acupuncture treatment from the same acupuncturist. The temperature of the treatment room will not be lower than 25 °C. Patients in the EA group will be treated with 0.25×25 mm and 0.25×40 mm disposable steel needles (Guizhou Andi Medical Instrument Co, Ltd).

Patients will be positioned supine after local skin disinfection with 75% alcohol wipes. The acupuncturist will insert needles into the GV20 (Baihui), DU24 (Shenting), EX-HN3 (Yintang), EX-HN18 (Anmian), EX-CA1 (Zigong), RN6 (Qihai), and RN4 (Guanyuan) acupoints as per the WHO Standard Acupuncture Point Locations [34]. KI3 (Taixi), KI7 (Fuliu), HT7 (Shenmen), and SP6 (Sanyinjiao) may also be used accordingly. KI3 and KI7 may be used for hot flashes; HT7 and SP6 are used for anxiety and depression. The needle is inserted with the double hand-needle insertion technique at a depth adjusted based on the standard permissible depth of insertion for each acupoint. The needle is then twirled, rotated, lifted, and thrust moderately until achieving the *deqi* sensation.

An electric stimulator will be placed on the pair of GV20 and EX-HN3 points with a continuous wave, 2.5 Hz, and 4-5 mA, for 30 minutes in each session. The other needles are retained for 30 minutes and manipulated for 10 seconds, twice every 10 minutes, with intermittent stimulation.

The needles will be removed with clean cotton balls to avoid bleeding. Each patient will receive 20 sessions over 8 weeks as follows: 3 per week in the first 4 weeks, every 2 days, with a 1-day interval; and 2 per week in the following 4 weeks, every 3 days, with a 2-day interval.

### Control Group

The 8-week CBT-I intervention consists of 1 face-to-face session weekly for a total of 8 sessions with a certified psychologist. Each session will be 45 minutes at a fixed time including setting objectives, learning skills, class activities, and assigning homework. The CBT-I protocol covers typical CBT-I components. The first face-to-face session will include an introduction by the therapist and CBT. After assessing the patient’s condition, the therapist will introduce the insomnia psychological patterns to stimulate the patient’s intention for the therapy. In the second session, the therapist will set sleep restriction and stimulation control strategies based on the first conversation and carry out targeted sleep education. Starting with the third session, the therapist will map the patient’s sleep diary and assess compliance to determine whether uptitration is allowed. Cognitive therapy for negative sleep beliefs will be performed from session 5. The principles of confidentiality, adherence, and autonomy will be followed throughout. At the end of each session, the therapist will assign homework to the participant and summarize the content of each treatment to provide skill consolidation and relapse prevention.

We will ensure patient confidentiality during the interview process and only mention the questions in treatment; no one shall disclose relevant information except the medical staff responsible for treatment. The client is expected to actively participate in the treatment process and, although there may be unforeseen events, we trust that the participant will adhere to the complete treatment process. Participants may leave the trial at any time during treatment. If, for some reason, the participant does choose to leave, there will be no effect on future treatment.

### Ethical Considerations

The ethics committee has reviewed the research plan, informed consent form, and other relevant clinical trial documents (2021-088). This trial can only be carried out with the written consent of the ethics committee. If unforeseen problems arise during the study, these should be reported to the ethics committee immediately. Patient priorities, experience, and preferences were not involved in the development of the research question and outcome measures, the design of this study, or the recruitment to and conduct of the study. The results will be not disseminated to study participants.

### Data Management

This experiment adopts electronic data management as described below and in the data management plan. The plan has been written by the data manager and will be carried out according to the described time, content, and methods. First, the data administrator will design the electronic case report form (eCRF) for the pilot study, a logic verification path is set according to the date verification plan, and the eCRF can be put into use only after passing a test and approval. The data in the eCRF are derived from the original record, and the data entry clerk enters the participant visit data as per the eCRF instructions. An inspector will then check the consistency of the eCRF data with the source data. All queries raised will be dealt with in a timely manner and addressed until the data is “clean.” After the database record is signed by the main researchers, bidders, statistical analysts, and data managers, the data administrator will lock the database. The eCRF of each participant will be saved as a PDF electronic document. After the data analysis, the data administrator will close the database.

### Quality Control

The trial will be conducted under the supervision of the Yueyang Hospital of Integrated Traditional Chinese and Western Medicine affiliated with the Shanghai University of Traditional Chinese Medicine. The clinicians in this trial have more than 10 years of clinical experience, and all the researchers will undergo training before the trial. A qualified clinical trial expert will be invited to monitor the study to identify any problems, examine the collected data, and control bias. Regarding quality control management, the data will be jointly managed through supervision, audit, and self-inspection. The procedures will be carried out in accordance with the relevant standard operating procedures.

### Outcome Measures

The primary outcome will be a mean change in total ISI from baseline to the end of the 8-week intervention. The validated ISI that has a concise design, good reliability, and has become a recognized tool for clinical researchers to evaluate the efficacy of insomnia will be used [35,36]. The ISI is a self-reporting questionnaire, and 7 self-rated items are included that evaluate the severity and impact of insomnia on patients. The total ISI score ranges from 0 to 28 points. The higher the score, the more serious the degree of insomnia, which is classified according to the total score (0-7 points represent clinically nonsignificant insomnia, 8-14 points represent subthreshold insomnia, 15-21 points represent moderately severe clinical insomnia, and 22-28 points represent severe clinical insomnia).

The secondary outcomes include the Menopause-Specific Quality of Life (MENQOL); Menopause Rating Scale; PSQI; Hamilton Depression Scale (HAMD); Hamilton Anxiety Scale (HAMA); hot flash score; and the level of estradiol, follicle-stimulating hormone, and luteinizing hormone in serum. Sleep architecture will be assessed using deep learning on polysomnograms (PSG). The ISI and MENQOL are measured at randomization and 4, 8, 12, and 20 weeks after randomization. The other outcomes are measured before and 8 weeks after randomization.

Menopause Rating Scale is a reliable method to measure the severity of menopausal compliance and contains 11 items in 3 dimensions (psychological, somatic-vegetative, and genitourinary systems). Each symptom is given a score of 0, 1, 2, 3, and 4 according to severity; the total is the sum of the scores of each item [37,38].

The PSQI scale is used to assess the quality of sleep during the previous month [39]. The scale consists of 9 questions, and the overall score ranges from 0 to 21. The higher the score, the worse the sleep quality. PSQI can be used to calculate patient sleep onset latency, time in bed, wake after sleep onset, total sleep time, sleep efficiency, and record the medicine used. Therefore, the combination of sleep quality and quantity can be used to assess sleep conditions and identify any sleep disorders.

The HAMD was developed by Hamilton [40] in 1960 to test the severity of depression in participants. The scale evaluation method is simple and is most used in clinical practice. The time frame for the assessment is the last 1 week. Two trained assessors jointly examine and score the patient by means of a conversation. A version with 17 items will be used in this study with scores of 0-7 regarded as having no depressive symptoms, 8-17 regarded as having mild depression, 18-24 regarded as having moderate depression, and 25-52 regarded as having major depression.

The HAMA scale uses conversation, observation, and independent grading to rate anxiety in the previous week. There are 14 items, and anxiety is scored as 0 to 4 (0: none; 1: mild; 2: moderate; 3: severe, and 4: very severe). The total ranges from 0 to 56 points, and the higher the score, the more severe the anxiety symptoms [41]. HAMA can also be used to assess medication use and the effect of psychological intervention.

Participants will complete validated hot-flash daily diaries for 7 days at baseline and 8 weeks after randomization, by recording the number of hot flashes and severity (mild, moderate, severe, or very severe). A composite score is generated by multiplying the number of mild, moderate, severe, or very severe hot flashes by 1, 2, 3, and 4, respectively, and summing the values into one score [42].

MENQOL is used to record the symptoms experienced by participants in the past month or to evaluate the clinical efficacy of quality of life in menopausal women. MENQOL includes 29 items, each with a score of 0-6; 0 means the symptom has no impact, and 6 means extreme impact. The scale records symptoms in the 4 domains such as vasomotor, somatic symptoms, psychological symptoms, and sexuality. The higher the score, the worse the patient’s quality of life [43].

PSG can objectively monitor sleep structure and efficiency, wakefulness, breathing, and periodic limb movements by recording the time off of lights; time in bed, total sleep time; sleep onset latency; wake-up time after sleep onset; total awake time from sleep onset to lights on; sleep efficiency; and sleep time in each period. Various physiological parameters such as electroencephalogram (EEG), electromyogram, electrooculogram, respiratory airflow, respiratory motion, and oxygen saturation are monitored [44,45]. Sleep staging will be performed by expert sleep technicians in nonoverlapping 30-second epochs as 1 of 5 stages such as wake, nonrapid eye movement (REM) stage 1, non-REM stage 2, non-REM stage 3, and REM.

### Safety Assessment

All adverse events (AEs) will be recorded during the trial and ranked as mild (does not affect the normal function of the patient), moderate (affects normal function to a certain extent), or severe (significantly affects normal function). Acupuncture syncope, pain at the acupuncture point, bleeding, hematoma, or infection that may occur during acupuncture; patients developing symptoms that are significantly unrelated to the current disease, including an exacerbation of a preexisting disease; abnormalities in medical detection or physical examination that require clinical treatment or further examination; and injury or accident are considered as AEs.

Any medical conditions (eg, vertigo) and accidents should be reported as 2 different AEs. Abnormalities found by laboratory tests, when related to another reported event or that require clinical treatment or further investigation, should be described in the remark’s column of the record and not treated as a separate AE.

### Statistical Analysis

If statistical demographic data and other baseline characteristics are continuous variables, they will be represented by the mean (SDs) and median (IQR) values. We will also include count and grade data for frequency and composition ratios. Concomitant medications will be categorized using the Anatomical Therapeutic Chemical code, percentage of patients with concomitant medications, and the incidence of AEs will be compared using the *χ*^2^ test or Fisher precision probability.

After 8 weeks of intervention, the change in the ISI score will be compared with the baseline. The subgroups and baseline are used as covariates to compare the differences between groups by analysis of covariance and the 2-sided 95% CIs of the least squares mean differences between groups. During each visit, the ISI score will be recorded, and mixed-effects model repeated measures will be used to compare the differences between groups. To compare 2 independent samples, a 2-tailed *t* test or nonparametric test will be used for continuous data, and chi-square or Fisher exact or nonparametric tests will be used for categorical data. The times and number of drugs that are taken by participants will be compared between groups using a rank sum test. When a covariance analysis is used for the primary efficacy indicator, missing values will be filled using the last observation carried forward method. When using the mixed-effects model repeated measures method for analysis, the missing data will not be filled. When an outlier is found, the main researcher, the applicant, and the statistician will discuss the processing method together before the database is locked. If necessary, the outlier value will be removed for sensitivity analysis.

## Results

Recruitment of participants for the study is presently underway. The inaugural participant was enrolled in January 2023, signifying the commencement of the recruitment phase. It is anticipated that the recruitment process will persist until January 2025, at which juncture data collection will commence.

## Discussion

### Principal Findings

This will be the first randomized, noninferiority, EA-controlled trial to study the efficacy and safety of EA compared with CBT for PMI. Considering that drugs may cause adverse reactions such as feeling drowsy, lightheaded, and dizzy, medications need to be limited to the lowest necessary dose and shortest necessary duration and nonpharmacological therapies are reasonable to use [18].

CBT-I has a longer duration of treatment and is suitable for patients with cognitive impairment [46]. It is offered in various forms such as one-on-one or in groups via telephone, the internet, or books. During the COVID-19 pandemic, some evidence-based psychotherapy was provided to medical staff to relieve mental pressure and prevent the occurrence of psychological disorders [47]. Web-based CBT has been shown to be effective in treating and preventing many stress-related symptoms among health care workers. Web-based CBT has also shown strengths in breast cancer survivors with clinically significant sleep problems [48].

Actigraphy (ACT) and PSG are commonly used to monitor sleep in clinical studies [49]. We have chosen PSG to monitor sleep as an objective efficacy index, as it makes a comprehensive estimation by monitoring various physiological parameters such as EEG, electromyogram, ECG, respiratory airflow, respiratory motion, and oxygen saturation while being manually analyzed by a sleep technician. Thus, sleep architecture is shown without bias. In addition, PSG is the gold standard for the assessment and diagnosis of sleep disorders [50]. However, PSG must be performed in the laboratory under the supervision of a relevant technician, and the discomfort of the electrodes and harnesses necessary for PSG monitoring brings about the “first night effect” [51], which also has a higher cost than other tests. Rather than recording EEG activity, ACT combines body movement frequency with the help of gravity sensors [52]. Although ACT is economical and simple to wear, it has a distinct drawback in that it may overestimate sleep time and underestimate wake time [53].

Women who are undergoing menopause transition seem to report more sleep difficulties. The content of some hormone changes dramatically in this stage, which may be associated with decreased melatonin synthesis and secretion. Progesterone reduces arousal and can have a sedative effect, while estrogen increases the turnover of the neurotransmitter norepinephrine and, subsequently, increases time spent in REM sleep and decreases REM latency [54]. Many of the menopausal symptoms (eg, hot flashes and night sweating) are precipitating factors for insomnia, which are in relation to the occurrence of poor sleep quality in menopausal women. Hot flashes are one of the most typical menopausal symptoms and are highly associated with awakening perimenopausal women in particular [55]. Repeated exposure to menopausal symptoms that cause wakefulness at night may perpetuate insomnia symptoms through conditioned arousal.

### Limitations

Our study protocol has some limitations. First, PSG brings discomfort to patients and reduces sleep quality. Although it is precise in the definition of wakefulness through accurate measurement with electroencephalography, PSG has insufficient validity for the assessment of wakefulness after sleep onset relative to wrist ACT. Second, we lack a follow-up assessment beyond 3 months. The durability of CBT-I is maintained 10 years after treatment [56]. Third, as our sample groups are going to be recruited from Shanghai, China, the inclusion of participants from different countries and regions would increase the persuasiveness of the trial results. The results may not be generalized to women who were taking certain medications, or who had certain gynecological surgeries (eg, hysterectomy and bilateral oophorectomy).

### Conclusions

Acupuncture and CBT-I have been recommended in the guidelines [57]. Indeed, if the efficacy of EA is noninferior to CBT, it could become an empirically assessed viable option specifically suited to the needs of women with PMI. The results of this study will provide knowledge for clinicians in the treatment of PMI.

## Appendix

Multimedia Appendix 1 SPIRIT (Standard Protocol Items: Recommendations for Interventional Trials) checklist.

## Abbreviations

ACT: actigraphy
AE: adverse event
CBT: cognitive behavioral therapy
CBT-I: cognitive behavioral therapy for insomnia
EA: electroacupuncture
eCRF: electronic case report form
EEG: electroencephalogram
HAMA: Hamilton Anxiety Scale
HAMD: Hamilton Depression Scale
ISI: Insomnia Severity Index
MENQOL: Menopause-Specific Quality of Life
MHT: menopausal hormone therapy
PMI: perimenopausal insomnia
PSG: polysomnograms
PSQI: Pittsburgh Sleep Quality Index
REM: rapid eye movement
SPIRIT: Standards for Reporting Interventions in Clinical Trials of Acupuncture

## Data Availability

The data sets generated in this study will be made available from the corresponding author. The study team will retain the exclusive use of the trial data until the publication of major outputs has been completed.
